# CXCL13 as a simple and promising blood biomarker for differentiating Sézary syndrome from mycosis fungoides and other confounding chronic inflammatory skin diseases

**DOI:** 10.3389/fimmu.2026.1804103

**Published:** 2026-04-10

**Authors:** Giulia Salvatore, Ylenia Aura Minafò, Nicoletta Croce, Francesca Passarelli, Cristina Cristofoletti, Maria Cristina Picchio, Laura Bonmassar, Stefania Madonna, Laura Mercurio, Alessandro Monopoli, Filomena Russo, Gaia Moretta, Enrico Scala, Stefania D’Atri, Maria Grazia Narducci

**Affiliations:** 1 Laboratory of Molecular Oncology, Istituto Dermopatico dell’Immacolata IDI-IRCCS, Rome, Italy; 2 Pathology Unit, Istituto Dermopatico dell’Immacolata IDI-IRCCS, Rome, Italy; 3 Laboratory of Experimental Immunology, Istituto Dermopatico dell’Immacolata IDI-IRCCS, Rome, Italy; 4 Department of Dermatology, Istituto Dermopatico dell’Immacolata IDI-IRCCS, Rome, Italy; 5 Clinical and Laboratory Molecular Allergy Unit, Istituto Dermopatico dell’Immacolata IDI-IRCCS, Rome, Italy

**Keywords:** cutaneous T-cell lymphoma, CXCL13, differential diagnostic marker, ELISA, IHC, RT-qPCR, Sézary syndrome

## Abstract

Sézary syndrome (SS) is a rare and aggressive type of cutaneous T-cell lymphoma (CTCL). Due to its rarity and marked biological heterogeneity, SS diagnosis is frequently delayed, as it requires the integration of multiple complex diagnostic tests interpreted by highly specialized biomedical figures. In this context, we investigated the expression of CXCL13—increasingly implicated in several inflammatory and neoplastic skin disorders—as a potential screening biomarker to discriminate SS from mycosis fungoides (MF), the most common CTCL subtype, and from clinically overlapping inflammatory skin diseases, i.e. atopic dermatitis (AD), psoriasis (PS) and eczema (EC). Real-time PCR analysis of CXCL13 mRNA expression in 51 samples showed a significant upregulation in peripheral blood mononuclear cells (PBMCs) from SS patients compared with MF, AD and healthy donors (HD). Protein-level analysis in a larger cohort (n = 142), including allergic and atopic EC patients, revealed significantly higher plasma CXCL13 concentrations in SS than in all other conditions and HD, as assessed by ELISA. Receiver operating characteristic (ROC) analysis confirmed the excellent performance of plasma CXCL13 as a differential marker (AUC = 93%). In contrast, CXCL13 immunohistochemical expression in skin biopsies was broadly detected across all conditions, limiting its diagnostic value. In conclusion, quantification of CXCL13 expression in PBMCs and, more robustly, measurement of its plasma levels by widely available techniques such as RT-qPCR and ELISA may represent practical screening tools to identify patients with suspected SS. These assays could facilitate earlier referral to specialized centres for confirmatory testing and prompt initiation of appropriate therapy.

## Introduction

Sézary syndrome (SS) is a rare leukemic variant of cutaneous T-cell lymphoma (CTCL), clinically characterized by erythroderma, lymphadenopathy and peripheral blood involvement. Unlike mycosis fungoides (MF), the most common form of CTCL, SS typically follows an aggressive clinical course and is associated with a poor prognosis, with reported 5-year survival rates ranging from 21% to 51%, depending on patient cohorts and therapeutic strategies (1, 2).

The diagnosis of SS remains notoriously challenging, as it requires the integration of clinical criteria (erythroderma), histopathological findings (epidermotropic T lymphocytes with cerebriform nuclei) and immunophenotypic abnormalities, including expansion of peripheral CD4^+^ T cells with a CD4/CD8 ratio >10, loss of pan-T-cell antigens such as CD2, CD7 and CD26, and downregulation of surface CD3, together with identification of a circulating malignant T-cell clone by flow cytometry (2).

An additional obstacle to obtaining a correct SS diagnosis depends on its initial clinical presentation, often mimicking chronic inflammatory skin diseases such as atopic dermatitis (AD), psoriasis (PS) and eczema (EC). This overlap often results in delayed diagnosis, particularly in non-specialized or resource-limited settings (3).

To facilitate SS recognition, several immunophenotypic and molecular markers have been proposed, including KIR3DL2 (CD158k), PD-1, PLS3, TWIST1, EPHA4, DNM3 upregulation and STAT4 downregulation. However, none of these markers has achieved routine clinical adoption, largely due to limited specificity, inter-patient heterogeneity, or insufficient assay standardization (4–10).

C-X-C motif chemokine ligand 13 (CXCL13), also known as B-cell-attracting chemokine 1 (BCA-1), is a homeostatic chemokine that binds CXCR5 and plays a key role in directing B cells and follicular helper T cells (Tfh) to germinal centres in secondary lymphoid organs and tertiary lymphoid structures (11, 12). Importantly, CXCL13 has and established association with CTCL entities with a T-FH phenotype, an emerging group that that appears to overlap with that of systemic cTFH lymphomas such as angioimmunoblastic T-cell lymphoma (13).

Accumulating evidence indicates that CXCL13 is ectopically expressed in a broad spectrum of autoimmune, inflammatory, lymphoproliferative and neoplastic skin diseases, suggesting a broader and likely pathogenic role in cutaneous disorders. In particular, CXCL13 has been associated with increased neutrophil survival (14), with the crosstalk between exhausted T-cells and cytotoxic T-cells (15), with disease severity and therapeutic response to anti-IL-23 treatment in psoriasis (16). In AD, *in vitro* models CXCL13 was also found to mediate cellular interactions among keratinocytes, fibroblasts and mast cells (17).

CXCL13 has been studied less extensively than other chemokines that are better characterized in CTCL (18). However, a landmark study from our group has shown that CXCL13 is overexpressed at the mRNA level in circulating neoplastic CD4^+^ lymphocytes from SS patients compared to healthy individuals, is detectable by immunohistochemistry (IHC) in skin lesions and lymph nodes from both SS and MF, and is present at higher plasma concentrations in SS compared with MF and other T-cell leukemias (19). Additional studies have confirmed CXCL13 expression in MF and AD skin biopsies, with a stronger association observed in SS by others (10). More recently, multi-omic analyses have shown that CXCL13^+^ malignant lymphocytes in MF are associated with B-cells forming tertiary lymphoid structures in skin-microenvironment. Authors also demonstrated, by analysing bulk RNAseq datasets, that CXCL13 is significantly overexpressed in malignant MF cells compared with benign inflammatory T cells infiltrating AD, PS and healthy skin (20). Building on these observations, we aimed to determine whether CXCL13 mRNA expression in peripheral blood mononuclear cells (PBMCs), its plasma concentration, or its tissue expression could serve as practical tools to support early SS diagnosis. This could favour timely initiation of effective treatments, with an improved outcome as demonstrated in SS patients undergoing extracorporeal photopheresis (ECP), a first-line immunotherapy for CTCL (21), or mogamulizumab, a recent monoclonal antibody drug used for relapsed or refractory CTCL (22).

Here, we show that CXCL13 mRNA expression in PBMCs and, more robustly, its plasma levels—but not its skin expression assessed by IHC—can reliably distinguish SS from MF and other confounding chronic inflammatory skin diseases, including AD, PS and EC. Quantification of CXCL13 by RT-qPCR and ELISA, easily achievable even in non-specialized centres, may therefore represent accessible screening approaches to guide clinicians toward more specific diagnostic investigations.

## Materials and methods

### Patients

This single-centre observational study analysed 142 plasma samples and 51 PBMCs samples, both fresh and cryopreserved, obtained from a total of 129 patients affected by different inflammatory and neoplastic skin diseases. Patients were enrolled between 2014 and 2025 at the IDI–IRCCS hospital. In addition, plasma and PBMC samples from 20 healthy donors (HD) were included as controls (**Table 1**).

**Table 1:** Demographic and clinical characteristics of patients enrolled in this study.

Disease	Clinical stage at sampling	N	Male: female (sex ratio)	Age (years, mean ± SD, range)	Therapy at sampling	N analysed by ELISA	N analysed by qRT-PCR	N analysed by IHC
SS	IVA1	38	17:21 (0.8)	68.0 ± 11.6 (44–85)	topical or systemic corticosteroids and antihistamine	38	13	4
MF	IB	5	3:2 (1.5)	71.2 ± 8 (61–83)	topical or systemic corticosteroids and antihistamine	5	4	1
MF	IIA	2	2:0 (n/a)	46 ± 21.2 (31–61)	ECP, interferon, methotrexate	2	1	1
MF	IIB	10	5:5 (1)	60.7 ± 15.7 (30-81)	ECP+interferon-α, bexarotene	9	6 †	1
MF	III	2	2:0 (n/a)	49.5 ± 2.1 (48-51)	ECP+interferon-α, brentuximab	1	2 †	1
AD	EASI: 32.78 ± 17.07 (5.8-72.3)	28	13:15 (0.9)	53 ± 16.2 (18-81)	washout period of at least four weeks	27	9 †	3
PS	PASI: 15.72 ± 9.01 (3.2-38.5)	24	16:8 (2)	54.4 ± 16.4 (24-76)	washout period of at least four weeks	20	6 ‡	4
Atopic EC	n/a	10	5:5 (1)	57.3 ± 17.7 (26-78)	topical corticosteroids and antihistamines	10	0	1
Allergic EC	n/a	10	5:5 (1)	40 ± 10.3 (28-60)	topical corticosteroids and antihistamines	10	0	1
HD	n/a	20	6:14 (0.4)	64.1 ± 21.1 (28-91)	n/a	20	10	2

†, 1 sample was analysed only by qRT-PCR.

‡, 4 samples were analysed only by qRT-PCR.

n/a, not applicable.

For SS and MF a mixed cross-sectional cohort design was adopted, including samples collected retrospectively between 2014 and 2020 and prospectively between 2020 and 2025. SS samples, all derived from patients who developed the disease *de novo* without any previous clinical history of MF, were collected at the time of diagnosis, during treatment with topical and/or systemic corticosteroids and antihistamines. MF samples were obtained both at early stages of disease (stages IB and IIA) and during disease progression (stages IIB and III), while patients were receiving the therapies detailed in **Table 1**.

For PS, AD and EC a retrospective cross-sectional study was carried out. PS and AD samples were collected from naïve or biologic-experienced patients enrolled between 2017 and 2025 after a washout period of at least four weeks from systemic therapies, whereas EC samples were collected between 2023 and 2025 while patients were undergoing treatment with topical corticosteroids.

The diagnosis of SS and MF was based on clinical, histological and biological criteria, in accordance with the WHO–EORTC classification of cutaneous lymphomas (2). The diagnosis of AD, PS and EC was based on established clinical criteria according to current international guidelines, disease severity in AD and PS was evaluated using validated scoring systems, namely the Eczema Area and Severity Index (EASI) for AD and the Psoriasis Area and Severity Index (PASI) for PS. The study was approved by the Ethics Committee of IDI–IRCCS and by the Comitato Etico Territoriale (CET) Lazio Area 5. Written informed consent was obtained from all participants in accordance with the Declaration of Helsinki (approval IDs: 4/CE/2015, 37/CE/2023, 474/CE/2016, 727/CE/2024, 09/CE/2023).

Demographic characteristics, clinical features and ongoing therapies at the time of sampling are summarised in **Table 1**.

### PBMCs isolation

PBMCs from SS, MF, AD, PS patients and HD were isolated by density gradient centrifugation using Lympholyte (Cedarlane), according to the manufacturer’s instructions. Cells were either immediately lysed for RNA extraction or cryopreserved in foetal bovine serum (FBS) containing 10% dimethyl sulfoxide (DMSO), stored in liquid nitrogen, and thawed when required.

### Real time PCR

CXCL13 gene expression in PBMCs was analysed in 13 SS, 13 MF, 9 AD, 6 PS patients and 10 HD. Total RNA was extracted using TRIzol reagent (Life Technologies), and DNA-free RNA was purified with the Direct-zol RNA Miniprep kit (Zymo Research) according to the manufacturer’s instructions. Complementary DNA (cDNA) was synthesised using the GoScript Reverse Transcription System (Promega).

Quantitative reverse transcription PCR (RT-qPCR) was performed using GoTaq qPCR Master Mix with CXR reference dye on a QuantStudio™ 5 Real-Time PCR System (Applied Biosystems), starting from 30 ng of cDNA. Relative gene expression was calculated using the ΔCt method, as previously described (23). Undetermined Ct values were set to the maximum cycle threshold (i.e. Ct=40). GAPDH was used as the housekeeping gene. Primer sequences are reported in **Supplementary Table 1**.

Mean ΔCt values from HD were used as reference for relative quantification. To account for inter-individual variability, mean fold-change values (RQ = 2^−ΔΔCt^) are reported together with RQmin and RQmax, calculated as follows: RQmin = 2^−(ΔΔCt + SD)^ and RQmax = 2^−(ΔΔCt − SD)^, where SD represents the standard deviation of the ΔCt values.

### Immunohistochemistry

Formalin-fixed, paraffin-embedded skin biopsies from patients with SS (n = 4), MF (n = 4), AD (n = 3), PS (n = 4), EC (n = 2) and from HD (n = 2) were retrieved from the archives of the IDI Pathology Unit. Histopathological classification was performed according to the European Organization for Research and Treatment of Cancer (EORTC) criteria (2, 24).

Immunohistochemical staining was carried out as previously described (17), with minor modifications. Briefly, antigen retrieval for CXCL13 was performed in citrate buffer (pH 6.0; Dako) by heating sections at 96 °C for 25 minutes. Endogenous peroxidase activity was quenched, and non-specific binding was blocked using Animal-Free Blocker (Vector, SP-5035) for 30 minutes. Sections were then incubated overnight at 4 °C with a goat polyclonal anti-CXCL13 antibody (AF801; R&D Systems) at a concentration of 2.5 µg/mL in 2% bovine serum albumin (BSA).

After washing, slides were incubated for 1 hour with a biotinylated anti-goat IgG secondary antibody (1:200), followed by incubation with the ABC reagent for 30 minutes. Signal detection was performed using diaminobenzidine (DAB; Dako). Slides were counterstained with haematoxylin, dehydrated and mounted for microscopic evaluation.

### ELISA

Peripheral blood samples from 38 SS, 17 MF, 27 AD, 20 PS, 20 EC patients and 20 HD were collected in lithium heparin tubes and centrifuged at 1700 × g for 10 minutes to collect plasma. Plasma aliquots were stored at −80 °C and thawed overnight at 4 °C before analysis.

Plasma CXCL13 concentrations were measured using a Human CXCL13 Quantikine ELISA kit (DCX130; R&D Systems), following the manufacturer’s instructions. For SS samples, an additional 1:2 dilution was performed prior to incubation. Absorbance was measured at 450 nm using an EnSight Multimode Plate Reader (PerkinElmer), with wavelength correction at 540 nm. CXCL13 concentrations were calculated from a standard curve generated by curve-fitting software. All samples were analysed in duplicate.

### Statistical analysis

Statistical analyses were performed using GraphPad Prism version 8 (GraphPad Software, La Jolla, CA, USA). Data distribution was assessed using the Shapiro–Wilk test. As at least one group in each comparison showed a non-normal distribution (p < 0.05), non-parametric tests were applied. Differences between multiple groups were analysed using the Kruskal–Wallis test followed by Dunn’s *post hoc* test, while pairwise comparisons were performed using the Mann–Whitney U test. Kaplan–Meier (KM) survival curves were analysed using the log-rank test.

## RESULTS

### CXCL13 is upregulated in PBMCs from SS patients compared to those with mimicking skin diseases

To investigate whether CXCL13 could represent a useful diagnostic biomarker for SS, we evaluated CXCL13 mRNA expression in PBMCs obtained at diagnosis from SS patients and from individuals affected by clinically overlapping skin diseases, including MF, AD and PS. HD were included as controls.

**Figure 1:**
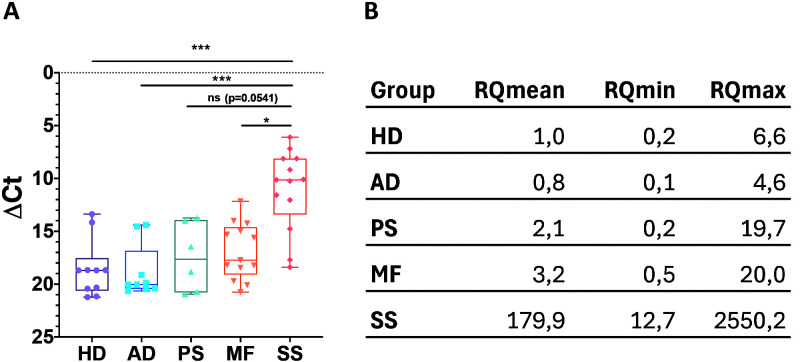
CXCL13 mRNA expression in PBMCs from patients with Sézary syndrome and other confounding skin diseases. **(A)** Box-and-whisker plots showing individual CXCL13 ΔCt values in PBMCs from patients with Sézary syndrome (SS), mycosis fungoides (MF), atopic dermatitis (AD), psoriasis (PS) and healthy donors (HD). ΔCt values were calculated using GAPDH as housekeeping gene. Statistical significance was assessed using the Kruskal–Wallis test followed by Dunn’s multiple comparisons test. ***p ≤ 0.001; *p ≤ 0.05. **(B)** Relative CXCL13 expression levels expressed as fold change (RQ) for each disease group relative to HD, calculated using the mean ΔCt value of HD as reference. RQmin and RQmax represent the minimum and maximum relative quantities obtained by adding or subtracting, respectively, the standard deviation of ΔCt values to ΔΔCt.

RT-qPCR analysis revealed a marked upregulation of CXCL13 transcripts in PBMCs from SS patients compared with all other groups (**Figure 1A**). CXCL13 expression was significantly higher in SS than in HD and AD (p < 0.001), with median ΔCt (IQR) values of 10.1 (8.1–13.4) for SS, 18.7 (17.5–20.6) for HD and 20.0 (16.8–20.4) for AD. A statistically significant difference was also observed between SS and MF patients (median ΔCt (IQR): 17.7 (14.6–19.1); p < 0.05). Although CXCL13 expression tended to be higher in SS than in PS, statistical significance was not reached, likely due to the limited sample size and high inter-individual variability within the PS group (**Figure 1A**).

Using the mean ΔCt value of HD as the reference, these differences translated into a marked increase in relative expression (RQ) in SS patients (mean RQ = 179.9), whereas only minimal increases were observed in MF (RQ = 3.2) and PS (RQ = 2.1), and no increase was detected in AD (RQ = 0.8) (RQmin and RQmax values are shown in **Figure 1B**).

Notably, all samples from non-SS groups displayed Ct values >30 or undetermined amplification, indicating very low transcript abundance. Consequently, although CXCL13 expression was clearly and significantly increased in SS, comparisons among non-SS groups were limited by the intrinsic sensitivity of the technique, which did not allow accurate quantification of very low transcript levels in MF and inflammatory skin diseases.

No differences in CXCL13 transcription levels were observed according to sex within each disease group (data not shown).

### CXCL13 immunohistochemistry does not distinguish SS from mycosis fungoides and other skin diseases

As CXCL13 expression in SS and MF skin lesions has not been comprehensively compared with that observed in other inflammatory skin diseases, we next evaluated CXCL13 protein expression by IHC in skin biopsies from patients with SS (n = 4), MF (n = 4), AD (n = 4), PS (n = 4), EC (n = 2), and HD (n = 2).

**Figure 2:**
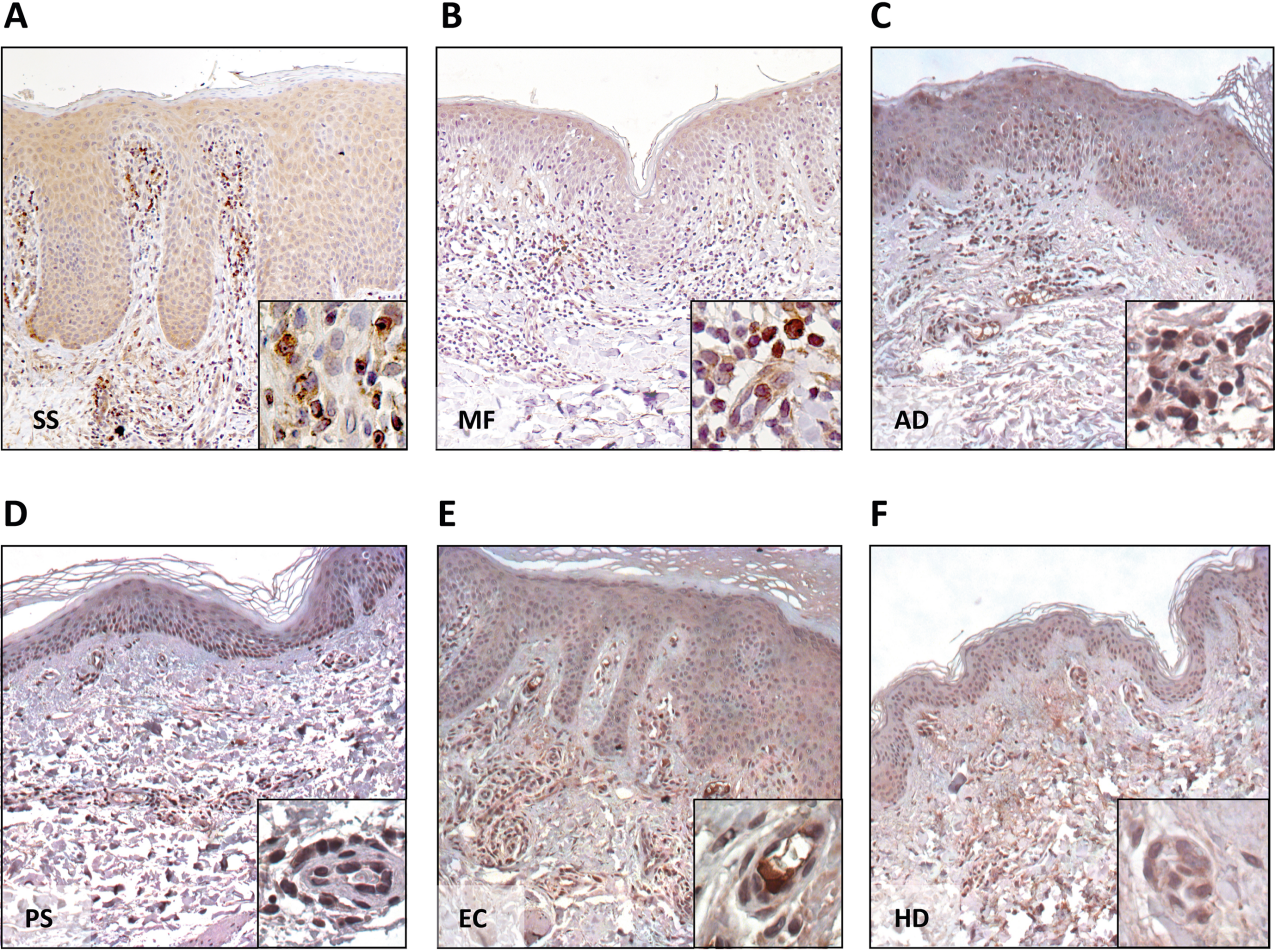
CXCL13 immunohistochemical expression in skin biopsies from Sézary syndrome, mycosis fungoides and inflammatory skin diseases. Representative immunohistochemical staining for CXCL13 in skin lesions from patients with Sézary syndrome [SS; **(A)**] and mycosis fungoides [MF; **(B)**], showing CXCL13 expression in endothelial cells and neoplastic lymphocytes infiltrating the dermis, frequently displaying a dot-like staining pattern. In atopic dermatitis [AD; **(C)**], psoriasis [PS; **(D)**], eczema [EC; **(E)**] and healthy donor skin [HD; **(F)**], CXCL13 immunoreactivity is mainly confined to endothelial cells and scattered non-neoplastic lymphocytes. Sections were counterstained with haematoxylin. Original magnification: ×10; inserts ×40.

CXCL13 immunoreactivity was detected in all SS and MF samples, although with variable intensity, in line with our previous observations (19). In both diseases, CXCL13 expression was mainly localised to endothelial cells and to neoplastic lymphocytes infiltrating the skin, which frequently exhibited a characteristic dot-like staining pattern (**Figures 2A, B**). In contrast, in AD, PS, EC and normal skin, CXCL13 expression was largely confined to endothelial cells and scattered non-neoplastic lymphocytes (**Figures 2C–F**).

Consistent with previous reports (10, 16), these findings indicate that CXCL13 is broadly detectable in this group of inflammatory and neoplastic skin conditions when assessed by IHC, thereby limiting its utility as a differential diagnostic marker when tested on skin biopsies.

### CXCL13 plasma levels distinguish SS patients from those with other confounding skin diseases

To assess whether circulating CXCL13 might provide greater diagnostic value than skin expression or PBMC transcript levels, we measured plasma CXCL13 concentrations by ELISA in an extended cohort of patients, also including individuals with atopic and allergic EC, and HD as controls.

**Figure 3:**
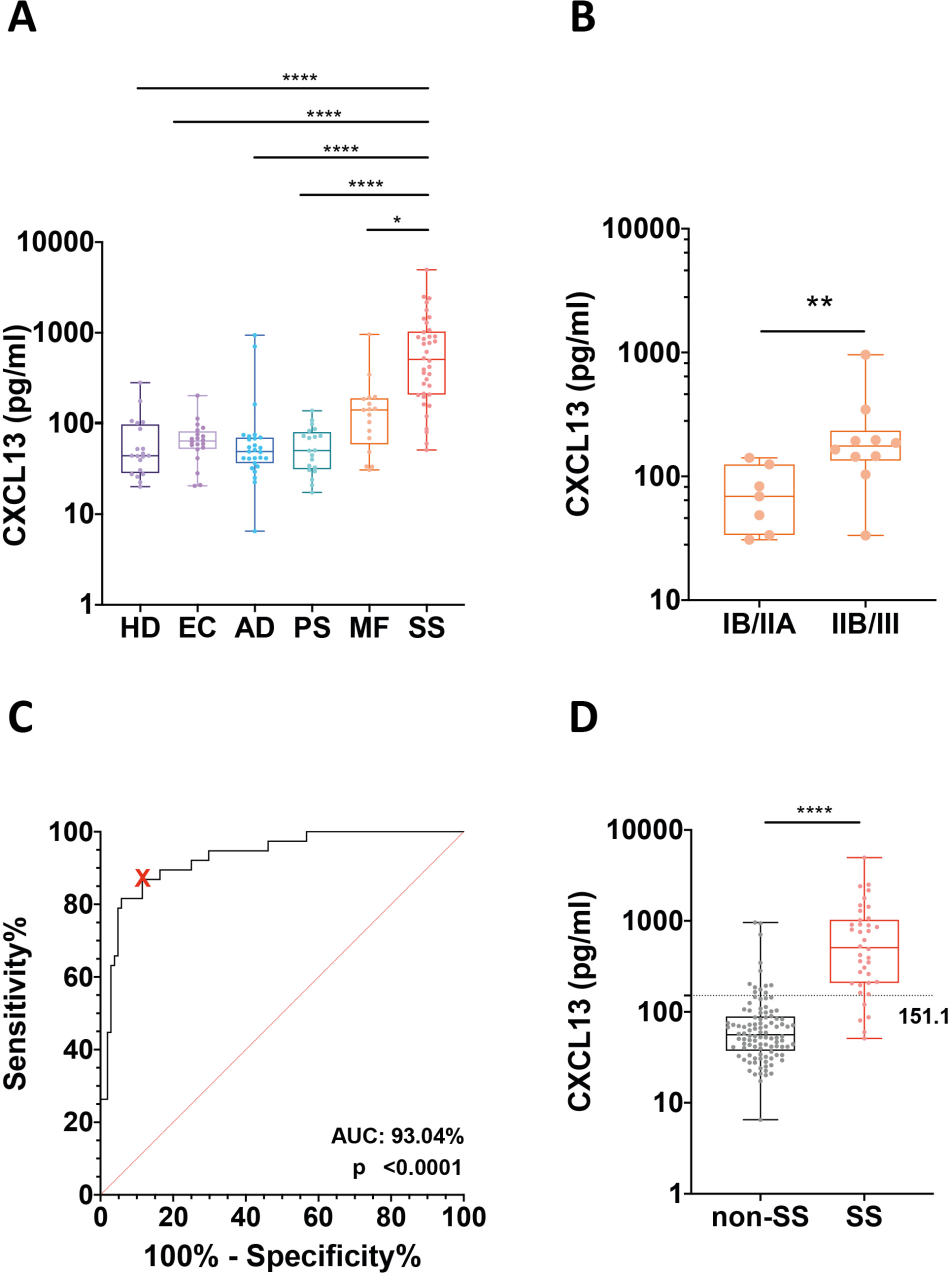
Plasma CXCL13 levels and diagnostic performance in Sézary syndrome. **(A)** Box-and-whisker plots showing individual plasma CXCL13 concentrations in patients with Sézary syndrome (SS), mycosis fungoides (MF), atopic dermatitis (AD), psoriasis (PS), eczema (EC) and healthy donors (HD). Statistical significance was assessed using the Kruskal–Wallis test followed by Dunn’s multiple comparisons test. **(B)** Plasma CXCL13 concentrations in MF patients stratified according to disease stage at sampling (stage IB/IIA *vs*. advanced stages IIB and III). Statistical significance was assessed using the Mann–Whitney U test. **(C)** Receiver operating characteristic (ROC) curve analysis evaluating the ability of plasma CXCL13 to discriminate SS patients from all non-SS individuals (MF, AD, PS, EC and HD). The red cross indicates the selected cut-off value (151.1 pg/mL) maximising sensitivity (87%) and specificity (88%). **(D)** Box-and-whisker plots showing individual plasma CXCL13 concentrations in SS (right) and non-SS (left) patients. The dotted line indicates the ROC-derived cut-off value. Statistical significance was assessed using the Mann–Whitney U test. ****p ≤ 0.0001; **p ≤ 0.01; *p ≤ 0.05.

Plasma CXCL13 levels were significantly higher in SS patients (median (IQR): 507.9 (207.7–1032) pg/mL) compared with HD and all other disease groups (**Figure 3A**). Median (IQR) values were 44.1 (28.4–97.8) pg/mL in HD, 64.0 (52.4–82.1) pg/mL in EC, 48.9 (36.5–69.9) pg/mL in AD, 50.3 (31.2–80.6) pg/mL in PS, and 141.2 (58.7–189.8) pg/mL in MF.

Correlation analyses performed in SS patients showed no significant association between plasma CXCL13 levels and tumour burden, assessed as the percentage of clonally expanded TCR-Vβ^+^ cells among CD4^+^ T cells, nor with overall survival calculated in months from the time of diagnosis. However, Kaplan–Meier analysis revealed a trend towards worse survival in SS patients with high CXCL13 plasma concentrations, defined as values above the cohort median (507.9 pg/mL), compared with those with lower levels (**Supplementary Figure 1A**). Importantly, these two groups did not differ in terms of tumour burden (**Supplementary Figure 1B**).

Among MF patients, early-stage disease (stage IB and IIA) (25) was associated with significantly lower plasma CXCL13 levels (median (IQR): 68.9 (33.5–125.0) pg/mL) compared with advanced stages (IIB and III; median (IQR): 175.5 (133.7–233.6) pg/mL; p < 0.01) (**Figure 3B**). Notably, only in MF patients did plasma CXCL13 levels significantly correlate with CXCL13 transcript expression in PBMCs (R² = 0.93, p < 0.0001; n = 11) (**Supplementary Figure 2**).

No sex-related differences in plasma CXCL13 concentrations were observed across disease groups, mirroring the findings obtained at the mRNA level (data not shown).

To further evaluate the diagnostic performance of circulating CXCL13, receiver operating characteristic (ROC) curve analysis was performed. CXCL13 plasma levels efficiently discriminated SS patients from all other groups, yielding an area under the curve (AUC) of 93.04% (p < 0.0001). A cut-off value of 151.1 pg/mL identified SS patients with 87% sensitivity and 88% specificity, corresponding to a positive predictive value (PPV) of 73.3% and a negative predictive value (NPV) of 94.8% (**Figures 3C, D**).

Importantly, plasma CXCL13 concentrations above this cut-off were observed even in SS patients with a moderate or low circulating clonal TCR-Vβ^+^ T-cell percentages, suggesting that CXCL13 may represent a valuable biomarker for SS identification at early disease stages, when blood involvement is limited and diagnosis is particularly challenging (**Supplementary Table 2**).

## Discussion

The diagnosis of SS remains particularly challenging, as no single, easily testable biomarker has yet achieved routine clinical adoption. Consequently, SS diagnosis is frequently delayed, especially given that this rare and heterogeneous disease can closely mimic a variety of chronic inflammatory skin disorders (3, 4, 6, 7). Although previous studies have reported CXCL13 expression as a feature of the aggressive CTCL microenvironment (7, 19, 20, 26–28), this chemokine has not yet been quantitatively assessed in a diagnostic setting nor systematically compared with other inflammatory skin diseases (10, 20), particularly using rapid and easily interpretable assays runnable in non-specialised centres.

In the present study, we demonstrate that CXCL13 transcript levels in PBMCs, assessed by RT-qPCR, are markedly increased in SS patients at diagnosis compared with healthy donors and patients affected by AD and MF, in whom CXCL13 expression was barely detectable.

Assessment of CXCL13 protein expression by IHC proved to be of limited diagnostic utility. As expected, routine histopathological evaluation revealed heterogeneous and semi-quantitative staining patterns across all diseases examined, confirming that CXCL13 IHC lacks sufficient discriminatory power when applied to skin biopsies (10, 16).

By contrast, the analysis of plasma CXCL13 concentrations in an extended cohort of patients revealed a clear and robust separation between SS and all other disease groups, including EC, AD, PS, MF and HD. The excellent diagnostic performance observed in ROC curve analysis further supports circulating CXCL13 as a reliable differential biomarker. Although the optimal cut-off value identified in this study (151.1 pg/mL) showed high sensitivity and specificity, independent validation in multicentre cohorts will be required before clinical implementation.

Notably, in keeping with the intended screening role of this biomarker, elevated plasma CXCL13 levels were detected even in SS patients with a moderate tumour burden. This observation suggests that CXCL13 quantification could be valuable in the early phases of the disease, when conventional blood-based criteria—such as increased circulating CD4^+^ T cells, elevated CD4/CD8 ratios (>10), CD4^+^/CD26⁻ ≥30% or CD4^+^/CD7⁻ ≥40%—may be insufficiently informative.

The lack of correlation between CXCL13 levels measured by ELISA or RT-qPCR and peripheral blood clonality in SS patients further suggests that the major source of this chemokine lies within tissue compartments preferentially colonised by malignant cells (2). In aggressive CTCL, these include the skin—where CXCL13^+^ tumour cells and CXCL13-driven tertiary lymphoid structures have been described (20) —and lymph nodes, in which neoplastic lymphocytes from SS patients exhibit strong CXCL13 immunoreactivity (19). The extensive involvement of both skin and lymph nodes in SS, even at disease onset, may therefore account for the markedly higher systemic CXCL13 levels observed in comparison with other inflammatory or neoplastic skin conditions.

While the single-centre design of this study may limit the generalisability of the findings, its strengths include the use of internationally recognised diagnostic criteria across all disease groups and the relatively large cohort of SS patients analysed at the time of diagnosis, before the initiation of disease-specific systemic therapies.

In conclusion, the present study supports the evaluation of CXCL13 expression in PBMCs by RT-qPCR and, more convincingly, the measurement of circulating CXCL13 levels by ELISA as innovative, rapid, non-invasive screening tools for the identification of patients with SS, even in non-specialised clinical settings. By facilitating earlier recognition of SS, these approaches may contribute to speed up diagnosis and, therefore, provide a therapy as early as possible with a positive impact on patients’ survival (21, 22).

## Data Availability

The raw data supporting the conclusions of this article will be made available by the authors, without undue reservation.
