# Supplementary material for: CXCL13 as a simple and promising blood biomarker for differentiating Sézary syndrome from mycosis fungoides and other confounding chronic inflammatory skin diseases
Source: Front Immunol. 2026 Apr 10;17:1804103. doi: 10.3389/fimmu.2026.1804103 (PMC13106335; doi:10.3389/fimmu.2026.1804103)
Supplement: Supplementary file 1 [file DataSheet1.docx]

Supplementary Material

## “CXCL13 as a simple and promising blood biomarker for differentiating Sézary syndrome from mycosis fungoides and other confounding chronic inflammatory skin diseases”

**Giulia Salvatore^1^, Ylenia Aura Minafò^1^, Nicoletta Croce^1^, Francesca Passarelli^2^, Cristina Cristofoletti^1^, Maria Cristina Picchio^1^, Laura Bonmassar^1^, Stefania Madonna^3^, Laura Mercurio^3^, Alessandro Monopoli^4^, Filomena Russo^4^, Gaia Moretta^4^, Enrico Scala^5^, Stefania D’Atri^1^, Maria Grazia Narducci^1*^**

^1^Laboratory of Molecular Oncology, Istituto Dermopatico dell'Immacolata IDI-IRCCS, Rome, Italy

^2^Pathology Unit, Istituto Dermopatico dell'Immacolata IDI-IRCCS, Rome, Italy

^3^Laboratory of Experimental Immunology, Istituto Dermopatico dell'Immacolata IDI-IRCCS, Rome, Italy

^4^Department of Dermatology, Istituto Dermopatico dell'Immacolata IDI-IRCCS, Rome, Italy

^5^Clinical and Laboratory Molecular Allergy Unit, Istituto Dermopatico dell'Immacolata IDI-IRCCS, Rome, Italy

**SUPPLEMENTARY FIGURES**


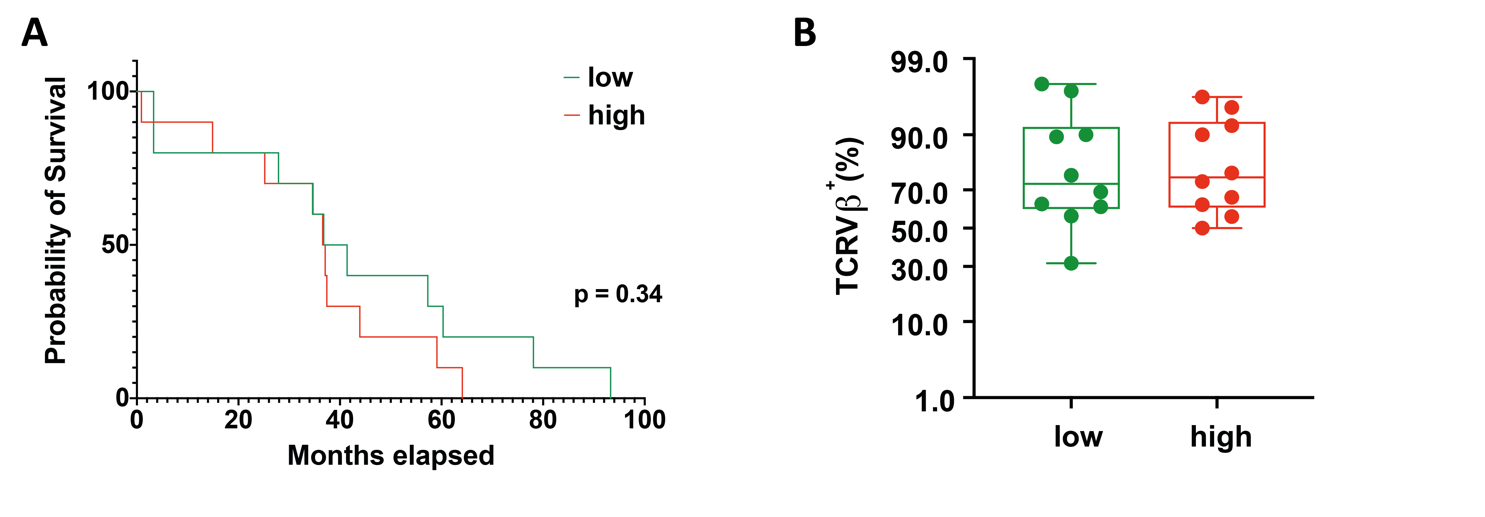


**Supplementary Figure 1 (A)** Kaplan–Meier survival analysis of patients with Sézary syndrome (SS) stratified according to plasma CXCL13 concentrations below (*low*) or above (*high*) the median value of the SS cohort (median, 507.9 pg/mL). Statistical significance was assessed using the log-rank test. **(B)** Box-and-whisker plots showing the circulating tumor burden for the same patients included in the Kaplan–Meier analysis, expressed as the percentage of patient-specific clonal TCR-Vβ⁺ cells among total CD4⁺ T cells.


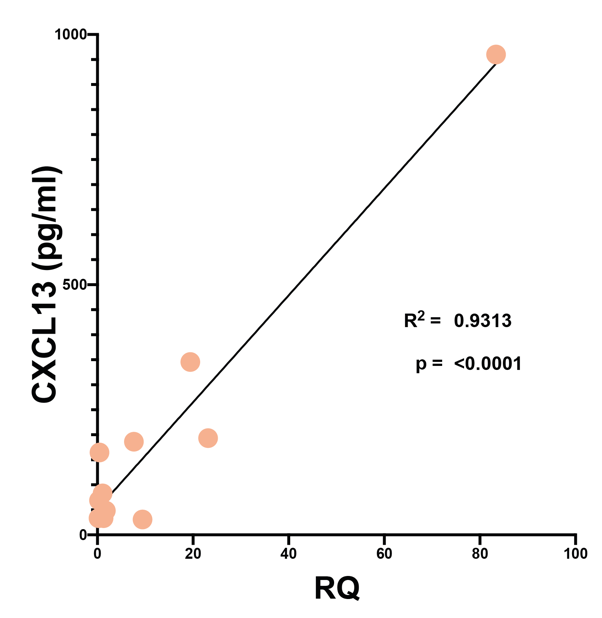


**Supplementary Figure 2** Correlation analysis between plasma CXCL13 concentrations and individual relative quantification (RQ) values for each patient, calculated using the mean ΔCt of healthy donors as the reference control.

**SUPPLEMENTARY TABLES**

**Supplementary Table 1**

**Primers used for CXCL13 quantitative real-time PCR (RT-qPCR) analysis**

| **Name** | **Sequence** |
| --- | --- |
| hCXCL13 Forward | 5' - TCC CTA GAC GCT TCA TTG - 3' |
| hCXCL13 Reverse | 5' - TAT CCA TTC AGC TTG AGG - 3' |
| hGAPDH Forward | 5′ - TCG GAG TCA ACG GAT TTG GT - 3′ |
| hGAPDH Reverse | 5′ - GAA TTT GCC ATG GGT GGA AT - 3′ |

**Supplementary Table 2**

**Clinical and molecular characteristics of patients with Sézary syndrome, including clonal TCR-Vβ rearrangement, circulating tumor burden (TB), and plasma CXCL13 levels measured by ELISA.**

| **Patient** | **Clonal TCR-Vβ+** | **Circulating TB †** | **CXCL13 (pg/ml)** |  |  |  |
| --- | --- | --- | --- | --- | --- | --- |
| SS77 | null | 61,5% | 80,48 |  |  |  |
| SS78 | null | 77,5% | 1019,19 |  |  |  |
| SS82 | 2 | 95,1% | 4954,14 |  |  |  |
| SS84 | 1 | 73,8% | 912,66 |  |  |  |
| SS85 | 13.6 | 97,0% | 197,09 |  |  |  |
| SS87 | null | 89,5% | 274,22 |  |  |  |
| SS88 | 3 | 56,6% | 306,85 |  |  |  |
| SS92 | null | 92,0% | 1069,93 |  |  |  |
| SS94 | 1 | 96,4% | 2403,13 |  |  |  |
| SS96 | 13.1 | 50,0% | 1775,46 |  |  |  |
| SS97 | 11 | 62,6% | 1484,59 |  |  |  |
| SS98 | 8 | 64,5% | 857,17 |  |  |  |
| SS100 | null | 90,0% | 156,61 |  |  |  |
| SS101 | null | 69,0% | 120,03 |  |  |  |
| SS102 | 17 | 90,0% | 1289,25 |  |  |  |
| SS103 | 5.2 | 31,5% | 206,18 |  |  |  |
| SS104 | 11 | 63,0% | 360,31 |  |  |  |
| SS106 | null | 66,4% | 803,31 |  |  |  |
| SS107 | 13.2 | 76,6% | 491,74 |  |  |  |
| SS111 | 13.1 | 85,0% | 895,67 |  |  |  |
| SS112 | null | 93,4% | 774,60 |  |  |  |
| SS113 | null | 82,5% | 1432,93 |  |  |  |
| SS114 | 17 | 23,5% | 907,35 |  |  |  |
| SS115 | null | 96,5% | 261,02 |  |  |  |
| SS116 | 5.1 | 97,6% | 349,80 |  |  |  |
| SS117 | null | 90,3% | 208,19 |  |  |  |
| SS118 | 5.1 | 76,0% | 421,83 |  |  |  |
| SS119 | 13.1 | 95,0% | 2497,81 |  |  |  |
| SS120 | 2 | 76,0% | 523,97 |  |  |  |
| SS121 | 22 | 76,0% | 87,15 |  |  |  |
| SS122 | null | 63,9% | 51,10 |  |  |  |
| SS123 | null | 56,3% | 755,62 |  |  |  |
| SS124 | 5.1 | 90,0% | 2173,50 |  |  |  |
| SS125 | null | 98,6% | 393,11 |  |  |  |
| SS126 | 4 | 36,0% | 212,97 |  |  |  |
| SS127 | null | 52,0% | 59,90 |  |  |  |
| SS128 | 1 | 13,0% | 162,12 |  |  |  |
| SS129 | 2 | 70,0% | 615,00 |  |  |  |
|  |  |  |  |  |  |  |
| † = percentage of clonally expanded TCR-Vβ^+^ cells among total CD4^+^ T-cells | | | | | | |
